# Developing monoclonal antibody therapies for measles could lead to adverse pathogen evolution

**DOI:** 10.1371/journal.pbio.3003701

**Published:** 2026-03-03

**Authors:** David A. Kennedy

**Affiliations:** Department of Biology and Huck Institutes of the Life Sciences, The Pennsylvania State University, University Park, Pennsylvania, United States of America

## Abstract

Monoclonal antibody therapies are being developed to treat measles in response to its recent resurgence. This Perspective argues that such therapies risk driving measles virus evolution in ways that might undermine the protection offered by vaccination.

## Teaser

Monoclonal antibody therapies are being developed to treat measles in response to its recent resurgence. These therapies risk driving measles virus evolution in ways that might undermine the protection offered by vaccination, outweighing potential benefits.

With vaccination rates against measles dropping in the United States and around the world, there has been a resurgence of measles cases [1] and, with it, a renewed interest in developing therapeutic treatments for measles. One of the clearest pathways for developing measles treatment is monoclonal antibody (mAb) therapy, and research along this route is already underway [2]. However, I would argue that the deployment of mAb therapy for measles (including cocktails of monoclonal antibodies) is a mistake that could potentially have irreversible and deadly consequences greatly disproportionate to any potential benefit.

The premise of mAb therapy for an infectious disease is to administer to a sick patient an antibody that binds to a known epitope (or antibody binding site) of the disease-causing pathogen, potentially inactivating or killing the pathogen. Such therapies show great promise for treating patients with otherwise difficult to treat diseases [3]. However, treatment runs the risk of driving pathogen evolution in such a way as to undermine the use of that same mAb in future patients. The evolutionary escape of mAbs has been seen before [4,5] and is an expected outcome of widespread mAb use. In many ways, mAb escape is similar to the evolution of antibiotic or antiviral drug resistance, yet there is one key difference: monoclonal antibodies almost always bind to the same epitopes as those recognized by natural or vaccine-induced immunity. The consequence of this is that pathogen evolution in response to natural or vaccine-derived immunity can undermine mAb therapy and vice versa. One example is COVID-19, where every mAb therapy that received emergency use authorization was pulled from the market shortly after introduction due to a loss of its efficacy that resulted from pathogen evolution in response to natural (and possibly vaccine-derived) immunity [6]. The big concern for measles, however, is that this process can potentially happen in reverse: that is, evolution in response to mAb therapy could in theory undermine vaccine protection. Measles vaccination has been highly efficacious in its current form for over 60 years. The disaster scenario would be if mAb-driven evolution undermined measles vaccination, which would undermine billions of people’s immunity. This is a plausible outcome of developing and deploying mAb therapies for measles (Box 1).

**Box 1. A simplistic model of measles vaccine escape risk.**

To develop a rough estimate of vaccine escape risk, one can ask how likely it is that a measles virus particle would be generated with enough mutations to escape vaccine protection. In practice, this can be approximated as $N{*\mu }^{M}$, where N is the number of virus particles, μ is the mutation rate, and M is the number of mutations that are required to escape vaccine-induced immunity.

For measles, at least five epitopes need to be mutated to undermine antibody-mediated vaccine protection (meaning minimally, M=5) [7]. Prior work has estimated the mutation rate (μ) of measles at 9 × 10^−5^ mutations per base per replication [8]. If we assume the number of virus particles per infected person (N) to be 1 × 10^10^, then the probability of a vaccine-escape variant being present in one infected person would be approximately 5.9 × 10^−11^, which can alternatively be interpreted as one vaccine-escape mutant being generated per 17 billion infections. If, however, monoclonal antibody (mAb) therapy drove evolution, such that the virus was now only four mutations away from vaccine escape (i.e., M=4), the probability of a vaccine-escape variant being present in one infected person would be approximately 6.6 × 10^−7^. This number can alternatively be interpreted as one escape mutant being generated per 1.5 million infections, which is fewer than the current number of yearly global measles cases. Note that in this depicted two-step process of pathogen evolution (i.e., mAb therapy escape followed by vaccine escape), in comparison to using a single mAb, cocktails of mAbs would be expected to delay the first step but accelerate the second step.

This simple model thus shows that the evolution of measles vaccine escape is plausible following mAb therapy escape, which itself can readily arise in the lab [4]. Notably, the above model depicts a worst-case scenario (neglecting biological details such as fitness costs and establishment probabilities); it should not be interpreted to state that mAb therapy will inevitably lead to vaccine escape, but merely that it is plausible. Regardless, given the outsized consequences, I would argue that measles mAb therapies should not be developed and deployed.

Vaccine-induced immunity to measles has so far been extremely robust against evolution: i.e., no variant of measles has ever emerged that is capable of sustainably spreading through a highly vaccinated population. This evolutionary robustness has been credited to the fact that measles vaccination induces a prophylactic, polyclonal antibody response that targets the measles virus at multiple epitopes simultaneously [9]. The diversity of this response ensures that no single mutation would allow the virus to escape the vaccine’s protection, and thus escape would require many changes to the measles virus (shown to be at least five) [7]. Importantly, none of these individual mutations are evolutionarily favored to spread in isolation from the others [7], and therefore the only way for a vaccine-escape variant to arise would be for a single virus particle to have many simultaneous mutations. This becomes exceedingly less likely to occur as the number of required mutations increases [9]. The evolutionary robustness of the measles vaccine, however, could be lost through the introduction of mAb therapy if such therapy were to create a stepping-stone towards vaccine escape.

In contrast to vaccination, mAb therapy is designed to target a single viral epitope. Single mutations may therefore be capable of conferring escape, which readily happens in the lab [4,7,8]. When escape mutations arise in treated host populations, these variants are likely to spread and undermine the functional life span of the mAb therapy. The bigger concern, however, is that it could also undermine the evolutionary robustness of the measles vaccine itself. This is because when the measles virus escapes a specific mAb therapy, it also escapes an epitope involved in vaccine protection. This evolution decreases the number of additional mutations required to escape vaccine protection and thus increases the chance that a mutant version of the virus will appear that is capable of spreading in vaccinated hosts. The risk of vaccine escape would be further compounded if multiple different mAb therapies were developed that targeted different epitopes—an active current area of research [2]. Once a mutant arises that can spread through vaccinated hosts, eliminating the use of mAb therapies would be ineffective at stopping the further spread of escape, since vaccine-acquired immunity itself would be able to drive further evolution [10]. In such an event, the billions of people in the world that have been vaccinated against measles would be at an increased risk for breakthrough infections, and this risk would only increase over time. In addition, there would be no obvious way back to the current status quo. Unless this risk can be shown to be extremely small, I would strongly advise against the development of mAb therapies to treat measles due to its potential risk to public health worldwide.

I am not advocating against mAb therapies in general. These therapies have promising potential for non-infectious diseases such as autoimmune disorders and cancers where no equivalent public health risks exist [3]. Moreover, even for mAb therapies that target infectious diseases, no equivalent risk exists when the therapy targets pathogen epitopes that already change over time or those that are peripheral to natural and vaccine-derived immunity. Likewise, the risks are low for mAb therapies that treat diseases against which immune protection tends to be short-lived or in situations where onward transmission is unlikely. However, when these mitigating features are absent, such as for measles, the risks of developing mAb therapies are quite large, and presumably outweigh the potential benefit. I therefore caution against the development and deployment of measles mAb therapies.

## Abbreviation:

mAb: monoclonal antibody
